# Non-linear dynamics of United States streamflow dataset

**DOI:** 10.1016/j.dib.2025.112092

**Published:** 2025-09-22

**Authors:** Krzysztof Raczyński, Katarzyna Grala, John H. Cartwright

**Affiliations:** Mississippi State University, Geosystems Research Institute, 2 Research Blvd, 39759, Starkville, MS, USA

**Keywords:** Streamflow, Dataset, Nonlinear, Chaos, Stochastic, Deterministic, United states

## Abstract

Comprehensive hydrological dataset containing daily, weekly, monthly, quarterly, and annual streamflow time series with corresponding fractal and chaos metrics for three flow regimes (maximum, average, and minimum) for 2899 gauging stations across the United States and Puerto Rico. Time series are available from January 1, 1970, to December 31, 2023, in five temporal resolutions in raw and filtered formats (where negative, zero, and missing data were interpolated). A suite of fractal and chaos metrics is included with data and contains Hurst exponents, detrended fluctuation analysis, multifractality, wavelet transform modulus maxima (with varying bands and modulus methods), sample entropy, recurrence quantification analysis, and Lyapunov exponents. Gauges were grouped via fuzzy C-means clustering into three dynamic-behavior groups, with a matrix of membership probabilities. Gauge metadata including corrected latitudinal and longitudinal coordinates is included. The resulting dataset includes raw and processed time series, computed fractal and chaos metrics, cluster assignments, and location metadata for each station. These data enable researchers and water resources managers to benchmark streamflow dynamics across scales, support the evaluation of hydrological models or resources, perform regional classification, and develop machine-learning applications.

Specifications Table

**Table:** 

Subject	Earth & Environmental Sciences
Specific subject area	Time-varying streamflow data across the United States and Puerto Rico with fractal and chaos characteristics.
Type of data	Table (.csv), Shapefile (.shp) Raw geospatial data (point gauge locations), filtered, filled, analyzed, and processed streamflow data (time series), and resulting characteristics (fractal data).
Data collection	Individual gauge raw streamflow data were collected from the USGS system. Values in ft³s⁻¹ were transformed to m³s⁻¹ and filtered to include only those containing <5 % missing or zero values in no more than one month of continuous series, with at least 50 years of data. Missing data were filled using linear interpolation. Daily, weekly, monthly, quarterly, and annual time series were built. Fractal and chaos characteristics were computed using the Python-based *pyenfra* package developed for this research and utilizing High-Performance Computing (HPC) infrastructure for large-set computation.
Data source location	Country: United States, Puerto Rico.
Data accessibility	Repository name: Time-varying Streamflow and Fractals Dataset for United States Data identification number: 10.4211/hs.44716e97517543889d2197aff6dd2cc3 Direct URL to data: https://doi.org/10.4211/hs.44716e97517543889d2197aff6dd2cc3 This dataset includes every gauge timeseries in daily, weekly, monthly, quarterly, and annual resolutions with raw format as well as missing data filled. Fractal dynamics and geospatial locations are included. Repository name: United States Streamflow Dynamics Dashboard Direct URL to data: https://www.arcgis.com/apps/dashboards/aa1975fa79f74131ac0c6950b66b38f5 Online dashboard proxy offering visual, map-level access to each gauge’s dynamic characteristics. The dynamic table in the left panel displays chosen values, and the user can customize their selection. The three-dot button in the top right corner of the table can be used to download tabular data. Repository name: USGS Current Water Data for USA Direct URL to data: https://waterdata.usgs.gov/nwis/rt? The Build Time Series tool allows generating raw data of daily streamflow values for a single selected station.
Related research article	Raczynski K., Grala K., Baran-Gurgul K., Cartwright J., Dyer J., 2025, Quantification of Nonlinear Dynamics and Chaos in Streamflow, *Journal of Hydrology*, (under review).

## 1 Value of the Data

• The dataset comprises long-term (1970–2023) streamflow records in five resolutions: daily, weekly, monthly, quarterly, and annual, and a comprehensive suite of fractal and chaos metrics for 2899 gauging stations across the United States (including Alaska, Hawaii, and Puerto Rico). These standardized, multi-scale descriptors of streamflow dynamics enable researchers to evaluate hydrological variability and complexity across diverse climatic and physiographic settings, benchmark non-linear behavior, and test new hydrological models under regulated vs. natural flow conditions.
• By providing both raw and processed time series alongside station metadata (verified latitude and longitude on river vectors), the data facilitate seamless integration into GIS‐based analyses, hydrological models calibration, and machine‐learning workflows. Users can link these metrics to environmental covariates (e.g., land use, climate indices) and explore drivers of hydrological behavior in varying environmental conditions.
• Fuzzy C-means clustering of gauges into three dynamic-behavior clusters, with membership probabilities, supports regionalization and classification studies. Other investigators can leverage these cluster assignments as priors for transfer learning or regional hydrological classification.
• All processing and analysis scripts are published in the open-source Python *pyenfra* package, promoting full reproducibility in other regions of the world. Researchers can reuse or extend these routines to compute additional complexity measures, apply alternative parameter settings, or analyze new gauging networks.

## 2 Background

Quantitative characterizations of streamflow complexity—spanning scaling behaviors, nonlinear dynamics, and multiscale variability—are essential for advancing catchment science and improving hydrological predictions. Yet, large-scale compilations of standardized fractal and chaos metrics remain scarce. To address this gap, we assembled daily streamflow records (January 1, 1970–December 31, 2023) for 2899 USGS gauging stations that met rigorous data‐completeness criteria. We computed a suite of complexity measures—including rescaled range, detrended fluctuation analysis (DFA), multifractal DFA, wavelet transform modulus maxima (WTMM), sample entropy, recurrence quantification analysis (RQA), and Lyapunov exponent estimation—and applied fuzzy C-means clustering to categorize dynamic behaviors of streamflow data across the United States. This work delivers the underlying dataset on time series, computed characteristics, and accompanying geospatial data, complementing our recent hydrological study [1] by ensuring transparency, enabling independent validation, and fostering new investigations into streamflow dynamics.

## 3 Data Description

### 3.1 Overview of streamflow characteristics

The dataset includes a suite of metrics describing the complexity and variability of streamflow across temporal scales. Table 1 summarizes each characteristic, its conceptual meaning in hydrological terms, and its typical interpretation. Details on the available data and methods used can be found in the Experimental Design, Materials and Methods section below.

**Table 1:** Description of streamflow non-linearity and chaos characteristics contained in dataset.

Metric	Description	Interpretation
Hurst exponent (RS)	Degree of long-term persistence or anti-persistence in flow fluctuations	>0.5 – persistent (high flows tend to follow high flows); <0.5 – anti-persistent (high flows tend to be followed by low flows)
Hurst exponent (DFA)	Degree of long-term persistence or anti-persistence in flow fluctuations	Same as RS with addition of >1 – indicating non-stationarity
Multifractality (MF-DFA slope)	Range of scaling behaviors across small vs. large fluctuations	Higher slope – greater variability in scaling between events
Wavelet modulus maxima slopes	Fractal scaling from wavelet decomposition	Sensitive to intermittent or bursty events
Sample entropy	Regularity/unpredictability of the time series	Low – more predictable; high – more irregular
RQA recurrence rate	Fraction of time the system revisits a prior state	High – stable, recurring patterns; low – more variable
RQA determinism	Predictability based on repeating patterns in phase space	High – strong deterministic structure; low – more stochastic
RQA entropy	Diversity of deterministic patterns	Higher – greater complexity in recurrence structure
Laminarity	Proportion of time the system stays in the same state	High – long steady states; low – rapid shifts
Trapping time	Average duration of steady states	Higher – longer stable flow conditions
Lyapunov exponent	Sensitivity to initial conditions (chaos)	>0 – chaotic; <0 – stable

### 3.2 Main repository: United States time-varying streamflow and fractals dataset

The available data is provided in multiple combinations that reflect the specific needs of the user. This section presents details of the main dataset directory and the visual proxy for accessing it.

**Fig. 1:**
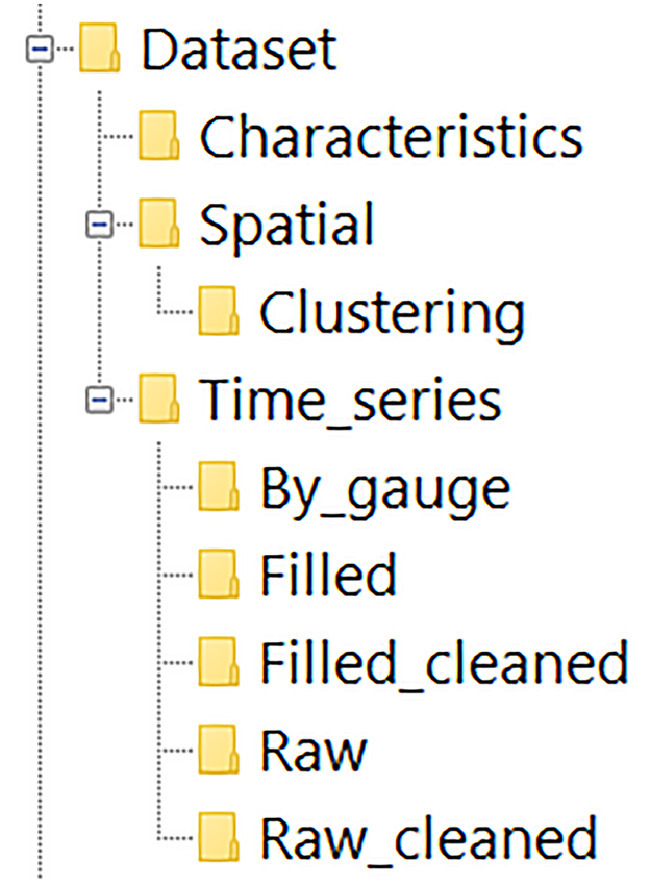
Folder structure in the dataset.

The main data repository is organized into folders (Fig. 1). On the main level, three folders are available:

• *Characteristics* — containing data tables on fractal dynamics of streamflow by gauge.
• *Spatial* — containing spatially referenced data for gauge locations.
• *Time_series* — containing raw and processed time series representing streamflow in each gauge in m³ s⁻¹ organized into subfolders based on the data type.

### 3.3 Characteristics folder

The *Characteristics* folder contains tabular data in the comma-separated values (.csv) format presenting fractal and chaos metrics for each gauge included in the dataset, as well as supporting files. For each aggregation resolution, i.e., weekly, monthly, quarterly, and annual, and for each flow regime: minimum, average, and maximum, tabular files are available (12 files in total). The naming convention for the files is as follows: aggregationResolution_flowRegime.csv

Each file contains 19 columns (Table 2).

**Table 2:** Column names and descriptions for characteristics files.

Column name:	Description:
fid	gauge identification number
Hurst_DFA	Hurst value by detrended fluctuation analysis
Hurst_RS	Hurst value by rescaled range method
Multifractality	MF-DFA alpha slope representing gradual change in generalized Hurst values
First Lyapunov	First Lyapunov exponent value
Entropy	Sample entropy
RecurrenceRate	RQA analysis recurrence rate
Determinism	RQA analysis determinism
Laminarity	RQA analysis laminarity
TrappingTime	RQA analysis trapping time
Max modulus Morlet low bands	Morlet wavelet with maximum value modulus, bandwidth 0.5, and center frequency of 1.0
Max modulus Morlet mid bands	Morlet wavelet with maximum value modulus, bandwidth 1.0, and center frequency of 1.5
Max modulus Morlet high bands	Morlet wavelet with maximum value modulus, bandwidth 1.5, and center frequency of 2.0
Mean modulus Morlet low bands	Morlet wavelet with mean value modulus, bandwidth 0.5, and center frequency of 1.0
Mean modulus Morlet mid bands	Morlet wavelet with mean value modulus, bandwidth 1.0, and center frequency of 1.5
Mean modulus Morlet high bands	Morlet wavelet with mean value modulus, bandwidth 1.5, and center frequency of 2.0
Normalized modulus Morlet low bands	Morlet wavelet with L2-normalized modulus, bandwidth 0.5, and center frequency of 1.0
Normalized modulus Morlet mid bands	Morlet wavelet with L2-normalized modulus, bandwidth 1.0, and center frequency of 1.5
Normalized modulus Morlet high bands	Morlet wavelet with L2-normalized modulus, bandwidth 1.5, and center frequency of 2.0

Additionally, two supporting files are available. The fields.txt contains the table fields description, the same as those provided in Table 2. The verbal_summaries.csv file provides human-readable descriptions of the streamflow dynamics by gauge and for each aggregation resolution and flow regime. An example of such a description is presented below:

“Average quarterly streamflows in this gauge exhibit persistent behavior (*H* = 0.52), and vague multifractal structure (MF-DFA slope: 0.021). Values of recurrence rate (0.2) indicate moderate recurrence, and the system is chaotic and noisy (Det=0.36). Entropy (1.8) indicates moderate complexity. Dynamics resemble fast switching, erratic system. Trapping time characterizing pace of transitions between states is 2.1.”

### 3.4 Spatial folder

This folder contains spatial data on dataset gauge locations in three formats:

• gauges.csv contains a comma-separated values file with five columns: *fid* (representing gauge identification number), *long* (presenting gauge longitudinal coordinate), *lat* (presenting gauge latitudinal coordinate), *station name* (providing name of the station according to USGS catalogue) and *drainage area* (watershed area in square miles, please refer to the Limitations section for data issues explanation). Coordinates are provided in the WGS84 (EPSG: 4326) Coordinate Reference System (CRS).
• gauges.gpkg contains the same gauge location information in GeoPackage format. This file is projected in the WGS84 (EPSG: 4326) Coordinate Reference System and ready for direct use in GIS software.
• gauges_shp.zip is a ZIP archive containing a shapefile (.shp) layer with supporting projection and reference files. This file is projected in the WGS84 (EPSG: 4326) Coordinate Reference System and ready for direct use in GIS software after extracting the archive.

All three files contain the same information on gauge locations with longitudinal and latitudinal coordinates in WGS84 CRS. Different formats are provided for user convenience.

The *Clustering* subfolder contains 12 files in comma-separated values format with a cluster membership probability matrix. Files have the following naming convention: aggregationResolution_flowRegime.csv

Each file contains five columns:

• fid – representing gauge identification number;
• p1 – probability (unscaled, i.e., 0–1) of belonging to the first spatial cluster;
• p2 – probability (unscaled, i.e., 0–1) of belonging to the second spatial cluster;
• p3 – probability (unscaled, i.e., 0–1) of belonging to the third spatial cluster;
• Highest probability cluster – cluster number selected on the highest probability value.

The fuzzy C-means clusters were forced with the same beginning state, which means cluster numbering between groups (e.g., monthly Q_min_ and annual Q_max_) is consistent. In general, the first cluster contains gauges characterized by the highest persistence and determinism, the second cluster contains gauges with the lowest persistence (or anti-persistence) and determinism, and the third cluster is intermediate. Please refer to the related research article for detailed characteristics [1].

### 3.5 Time_series folder

This folder contains time series datasets for both raw and processed datasets. All values are provided in m^3^ s^-1^. Total tables (for all gauges together) are available in comma-separated values format in the following subfolders, describing data processing method:

• *Raw* - raw dataset is the original non-processed data with no missing (“NaN”) or zero (“0”) and negative values altered;
• *Raw_cleaned* - raw dataset with zero (“0”) and negative values transformed to missing (“NaN”);
• *Filled* - processed *Raw* dataset with missing (“NaN”) values interpolated; zero values (“0”) and negative values are not altered;
• *Filled_cleaned* - processed *Raw_cleaned* dataset with all zero (“0”), negative, and missing (“NaN”) values interpolated.

Each of these folders contains the same data structure, with 13 files corresponding to different aggregation resolutions and flow regimes, with the following naming convention: aggregationResolution_flowRegime.csv

The exception is the daily flow dataset, which is not aggregated (raw data) and presents no specific flow regime.

• *By_gauge* folder contains separate datasets for each included gauge organized by identifier. Each individual gauge data is packed in a .tar.gz archive and requires extraction. Within the individual dataset directory (after extracting), there are 52 comma-separated values files with the naming convention as follows:

aggregationResolution_flowRegime_datasetType.csv where *aggregationResolution* is a timescale the data was aggregated in: annual, quarterly, monthly, and weekly, as well as non-aggregated daily data; *flowRegime* is a description of aggregation method for each timescale: min, max, and mean; and *datasetType* corresponds to method of data processing as described above: raw, raw_cleaned, filled, filled_cleaned.

The above structure description is also presented in the supporting file description.txt located in the *Time_series* folder. An additional *data_log.csv* tabular file is provided. This file contains information on:

• number of zero or negative values contained in the Raw set (filed: “0 values Raw set”),
• number of missing values in the Raw set (field: “NaN values Raw set”),
• number of total missing values in the Raw_cleaned set (field: “NaN values Raw_cleaned set”),
• number of values that were interpolated in the Filled set (field: “0 values Filled set”),
• number of values that were interpolated in the Filled_cleaned set (field: “0 values Filled set”), and
• indexes of values that were filled (field: “Filled values Filled_cleaned set).

### 3.6 Visual access: United States streamflow dynamics dashboard

**Fig. 2:**
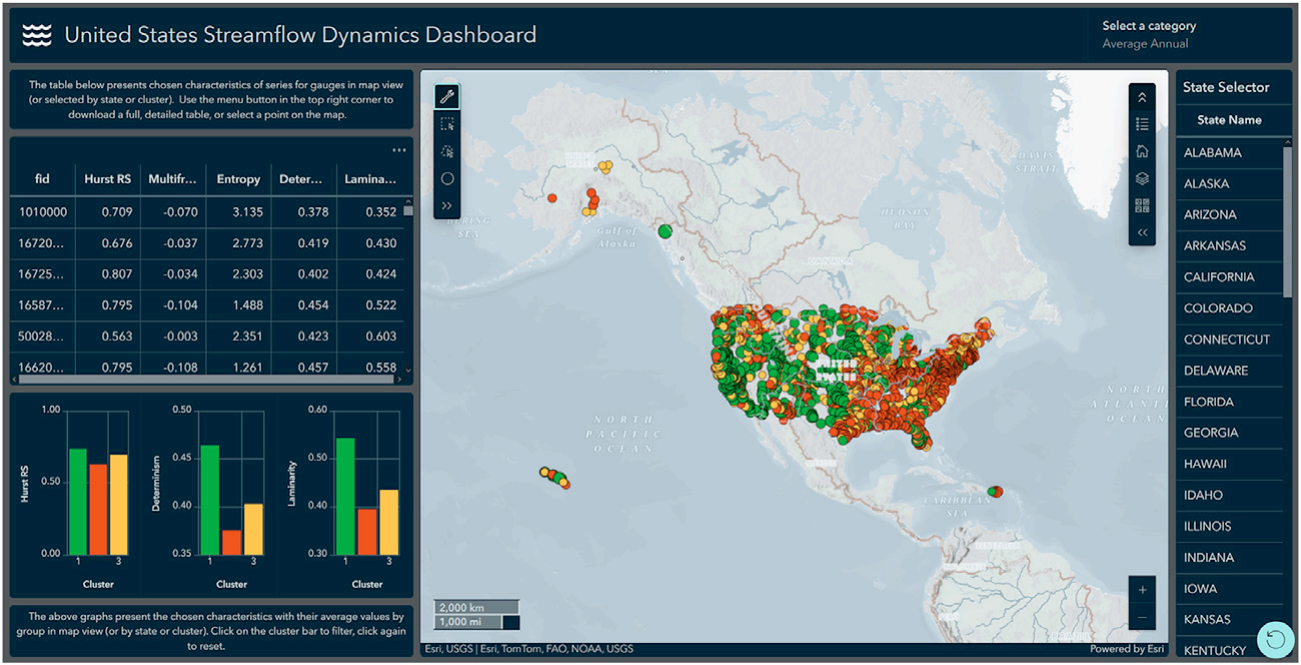
Visual proxy for data download in the form of online dashboard default view.

The online dashboard proxy offers visual, map-level access for each gauge dynamics characteristic. In the default view, the user is presented with dynamic data for average annual streamflow, and this category can be changed using the category selector (Fig. 2, upper right corner). Each gauge is colored based on the cluster membership (based on the highest probability), and the size of the point corresponds to the probability value.

**Fig. 3:**
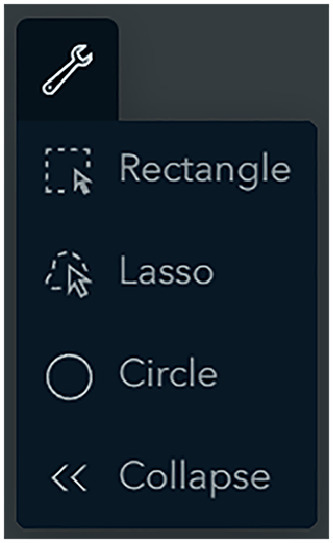
Custom selection tools menu.
• Interactive graphs, available in the lower left part of the dashboard, display the mean value of selected characteristics by group. This information is adjusted to the map extent shown in the main window. Zooming in, moving the map or making a selection on the map adjusts the filter criteria displayed. Graph bars can be selected to apply specific cluster filters.
• The interactive table, available on the left, similarly to graphs, adjusts the content based on the main map view and all the above-mentioned selections if applied. Additionally, by selecting a row (or rows) in the table, the user can add custom filtering.

Individual characteristics can be displayed by clicking the gauge point. The dashboard offers multiple ways of point selection for data download:

• The state selector, available on the right side of the map, allows filtering points by state by selecting it from the list.
• Selection tools allow for custom gauge (or gauges) selection by drawing a shape on the map (Fig. 3).

Each selection applies to the current category displayed under “Select a category” in the upper right corner.

**Fig. 4:**
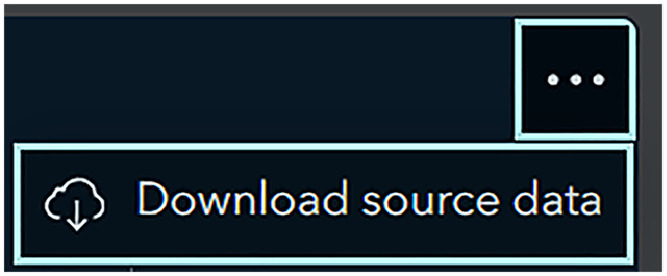
After the view is adjusted or a selection is made, the interactive table allows to download the unabridged table in comma-separated value format.

When gauges are selected or the map view is adjusted, the ellipsis button in the top right corner of the interactive table can be used to download tabular data (Fig. 4). The interactive table presented in the dashboard contains selected key characteristics; however, complete information on all the available variables is available in the downloaded file (comma-separated value format).

## 4 Experimental Design, Materials and Methods

### 4.1 Data acquisition and preprocessing

Daily mean streamflow records for 3135 USGS gauging stations were retrieved from the USGS Current Water Data repository [2] for the period January 1, 1970, through December 31, 2023. Stations with >5 % missing or zero flow values were excluded from analysis. Of the remaining 2899 stations, internal data gaps were filled by linear interpolation. Series were aggregated using three metrics to represent different streamflow regime conditions: maximum, average, and minimum values, in weekly, monthly, quarterly (3-month), and annual (12-month) resolutions. For each aggregation period the “maximum” flow regime represents the single largest daily mean discharge value within that period, and the “minimum” regime represents the single smallest daily mean discharge within the period. The “average” regime is the arithmetic mean of all daily mean discharges in the period. This approach preserves the extremes actually observed in the underlying daily record for that period, rather than averaging over peaks or troughs, and was applied consistently for all gauges and temporal resolutions. Gauge latitude and longitude were obtained from the USGS National Water Information System [2]. The final dataset spans gauges across the contiguous United States, Alaska, Hawaii, and Puerto Rico.

### 4.2 Computational environment

All analyses were performed in Python 3.11, employing the developed open-source *pyenfra* package [3]. High-performance computing (HPC) infrastructure was used for computation, employing a node with dual 20-core Xeon Gold 6148 processors and 384 GB of RAM. Time series were analyzed over 20 logarithmically spaced scales (minimum window size *n* = 10), with at least 10 segments per scale to ensure robust statistics.

### 4.3 Non-stationarity and chaos characteristics

The analytical methods implemented in this dataset have a well-established presence in hydrological and hydro-climatological research. Examples include studies of long-term persistence in streamflow [4,5], detection of multifractal scaling in precipitation and runoff [6], and nonlinear dynamics in river discharge [7,8]. A summary of methodologies and values interpretation of these characteristics can be found below.

### 4.4 Rescaled‐range (RS) analysis for Hurst exponent

The Hurst exponent was computed using the methodology introduced by Hurst [9]. Each time series *X*(*t*), *t* = 1, …, *N*, was divided into non-overlapping windows of length *n*. Within each window, the cumulative deviation was computed:

(1) $$Y\left(t\right)=\sum_{i=1}^{t}\left(X\left(i\right)-\bar{X}\right)$$

where:

$\bar{X}$ – sample mean.

The range and standard deviation yield the rescaled range:

(2) $$RS=\frac{R\left(n\right)}{S\left(n\right)}$$

where:

*R*(*n*) – range, as *R*(*n*) = max[*Y*(*t*)] − min[*Y*(*t*)]

*S*(*n*) – standard deviation.

Hurst exponent *H* is estimated by linear regression on log*RS* vs. log*n*. The value of H indicates the nature of the correlations in the data: *H* > 0.5 implies persistence, *H* = 0.5 corresponds to a random walk, and *H* < 0.5 suggests anti-persistence. A 5 % RS confidence interval around the random-walk hypothesis (*H* = 0.5) was calculated following the Weron method [10]. This method is used in hydrology to detect long-term persistence in streamflow records [4].

### 4.5 Detrended fluctuation analysis (DFA)

The series was first integrated as in RS analysis, then divided into non-overlapping segments of length *n*. In each segment, a local polynomial trend *y_n_*(*t*) was fitted and subtracted to obtain detrended fluctuations [11]. The root-mean-square fluctuation function:

(3) $$F\left(n\right)=\sqrt{\frac{1}{N}\sum_{t=1}^{N}{\left[Y\left(t\right)-y_{n}\left(t\right)\right]}^{2}}$$

was computed for each scale *n*, and its power-law scaling *F*(*n*)∼*n^α^* yielded the scaling exponent *α*. Interpretation of *α* follows Hurst thresholds (i.e., *α*=0.5 for white noise) with the addition of values *H* > 1, where *H* = 1 represents pink noise, *H* > 1 indicates non-stationarity tending toward super-diffusive behaviour (for *H* > 1.5) and Brownian noise (*H* = 1.5). Weron’s method for 5 % DFA confidence intervals was used for each *α* [10]. DFA have been successfully used to examine persistence in hydro-climatic time series [5].

### 4.6 Multifractal DFA (MF-DFA)

Extending DFA, the *q*th-order fluctuation function [5,12]:

(4) $$F_{q}\left(n\right)={\left\{\frac{1}{N_{n}}\sum_{v=1}^{N_{n}}{\left[F^{2}\left(v,n\right)\right]}^{q/2}\right\}}^{1/q}$$

where:

*F*^2^(*ν,n*) – variance in the *ν*-th segment of length *n*

*N_n_* – number of segments was computed over the same scales. For *q* = 0, logarithmic averaging was used. The generalized Hurst exponent *h*(*q*) was obtained from the scaling *F_q_*(*n*)∼*n^h^*^(^*^q^*^)^; the *q*-dependence of *h*(*q*) characterizes multifractality (monofractality if *h*(*q*) is constant). For each time series, the scale parameter (*n*) values were determined adaptively to accommodate series length while ensuring robust statistics. The minimum scale was fixed at 10 data points, and the maximum scale was set to the largest integer allowing at least 10 non-overlapping segments. Between these bounds, 20 logarithmically spaced *n* values were generated, identical across all flow regimes (minimum, average, maximum) and temporal resolutions (daily, weekly, monthly, quarterly, annual). This approach preserves comparability between stations of varying record lengths while preventing bias from underrepresented large scales. The same procedure was applied in all MF-DFA computations, with *q* values fixed at 11 equally spaced points in the range −5 to 5.

### 4.7 Note on Hurst exponents

Three approaches were applied to estimate long-range persistence in streamflow series: the classical rescaled range (RS) method, detrended fluctuation analysis (DFA), and multifractal DFA (MFDFA). RS and DFA, while differing in detrending procedures, produce broadly comparable estimates for persistent/anti-persistent behaviour whereas MF-DFA allows for scale-dependent variation and is used to evaluate multifractality rather than to define a single H value. To ensure comparability across gauges and minimize methodological bias both RS and DFA values are provided with related 5 % confidence intervals in addition to MF-DFA outputs characterizing multifractal spread. This approach balances robustness against noise with sensitivity to persistent/anti-persistent behaviors, enabling consistent spatial and temporal comparisons across the network.

### 4.8 Wavelet transform modulus maxima (WTMM)

Fractal dimension was assessed via continuous wavelet transform (CWT) multiscale transition thresholding. The CWT:

(5) $$W_{f}\left(a,b\right)=\frac{1}{\sqrt{a}}\int_{-\infty }^{\infty }f\left(x\right)\psi \left(\frac{x-b}{a}\right)dx$$

was computed using three Morlet wavelets in the following combinations:

• bandwidths of 0.5, 1, 1.5
• center frequencies of 1, 1.5, 2

Modulus maxima in three combinations: maximum, mean, and L2-normalized were used, and lines across scales *a* yielded the partition function:

(6) $$Z\left(q,a\right)=\sum_{i}{\left|W_{f}\left(a,b_{i}\right)\right|}^{q}$$

from which the multifractal spectrum *τ*(*q*) was estimated [13,14]. The WTMM method in hydrology is used to detect multi-scale intermittency [6].

### 4.9 Lyapunov exponent and embedding dimension

The largest Lyapunov exponent *λ* was estimated using the Eckmann algorithm [15]; *λ*>0 indicates chaos. Minimum embedding dimension was determined by the false nearest neighbors method [16], selecting the smallest dimension at which the proportion of false neighbors falls to near zero values (1 % threshold). Largest Lyapunov exponent estimation via Eckmann et al. (1986) [15], with embedding dimension following Kennel et al. (1992) [16].

### 4.10 Sample entropy (SampEn)

Complexity was quantified by sample entropy [7,17]:

(7) $$SampEn=-ln\frac{A}{B}$$

where *B* is the number of pairs of embedding vectors of length *m* = 2 within tolerance *r* = 0.2*σ*, and *A* is the count for length *m* + 1. Lower *SampEn* indicates greater regularity.

### 4.11 Recurrence quantification analysis (RQA)

For recurrence quantification analysis [18,19], phase-space embeddings *x_i_*=[*x*(*i*),*x*(*i*+*τ*),…, *x*(*i*+(*m* − *1*)*τ*)] were constructed. Recurrence plots *R_i_*_,_*_j_*=Θ(*ε*−∥*x_i_*−*x_j_*∥) used a threshold *ε* set at the 10 % percentile of distances. RQA was performed on phase-space reconstructions of the normalized streamflow time series for each flow regime and temporal resolution. The reconstruction used the same embedding dimension (*m*) and delay (*τ*) as in the largest Lyapunov exponent estimation: *m* determined via the false nearest neighbors method [16] and *τ* determined by the first minimum of the average mutual information function. Using consistent embedding parameters across RQA and Lyapunov analysis ensures that all nonlinear dynamic metrics describe the same underlying phase-space geometry. Standard RQA metrics were computed: recurrence rate (*RR*), determinism (*Det*), entropy (*Entr*), longest diagonal (*L_max_*), divergence (*Div*), laminarity (*Lam*), and trapping time (*TT*). These quantify memory, predictability, and regime dynamics.

### 4.12 Note on the entropy characteristics

In this dataset, two entropy measures are provided. Sample entropy (*SampEn*) is a time-domain statistic quantifying the irregularity of a one-dimensional series, based on the conditional probability that sequences of length *m* match again at length *m + 1* within a given tolerance (*r*). RQA entropy is computed from the distribution of diagonal line lengths in the recurrence plot, capturing the diversity of deterministic patterns in reconstructed phase space. While both measures increase with system complexity, they are derived from different mathematical frameworks and are not interchangeable.

### 4.13 Spatial clustering and statistical analyses

To capture regional flow-regime patterns, fuzzy C-means clustering was applied to a reduced parameter set obtained via principal component analysis (PCA). PCA retained metrics (*Hurst, α, h*(*q*), *SampEn, Det, Lam, TT*, and wavelet-derived measures) explaining 90 % of variance. Pairwise Spearman’s rank correlations guided exclusion of redundant metrics. FCM (fuzzification parameter *m* = 2.0; convergence threshold 10^−5^) partitioned gauges into three clusters, producing membership degrees that reflect transitional hydrological regimes.

A significance level of *α* = 5 % was used in all tests.

## Limitations

Missing, zero, and negative streamflow values constitute the first limitation of the dataset. Linear interpolation of in-series gaps may smooth high-frequency variability, potentially biasing complexity metrics, and further incomplete early records (missing start-of-series months) may affect cross-site comparability. To address that, the provided dataset contains both raw and filled sets with an assisting filing log for users to apply the data most suited for their needs.

The presented dataset begins at the daily scale, with weekly to annual aggregations, to maximize spatial coverage and record length under the data-completeness criteria applied. While daily resolution captures a wide range of hydrological variability, it does not resolve short-duration peaks characteristic of flash floods in small basins. Nationally consistent sub-daily (hourly or finer) streamflow records are generally shorter and more fragmented, therefore they are not in the scope of this dataset.

Used parameter values for fractal and chaos analyses (e.g., scale ranges, entropy tolerances, wavelet scales) follow standardized protocols but may not be optimal for all catchments due to localized specifics. The developed *pyenfra* environment allows for parametric modifications in case specific parametrization is needed.

Clustering into three fuzzy C-means groups simplifies the continuum of hydrological behaviors and may overlook finer regional heterogeneity. The three cluster levels were chosen experimentally, based on the entire study domain fit; however, the membership probability matrix is provided for individuals to determine localized grouping.

Finally, the drainage area provided in the spatial data represents the values “as available” in the USGS catalogue. Users should be aware and consider the following limitations when handling this information:

• Some drainage area information might be missing or invalid (e.g. 0 values);
• Some catchments contain delineation errors related to gaps, overlaps or geometry error (e.g.: https://waterdata.usgs.gov/monitoring-location/USGS-09217000). Users should refer to USGS gauge information (https://waterdata.usgs.gov/) for reference on used drainage area;
• Drainage areas are delineated using HUC extents and catchment outlets are not gauge aligned.

Moreover, many gauging stations in the United States are located downstream of dams, reservoirs, or other flow-regulating structures. Such regulation can dampen or amplify certain flow variations, alter scaling behaviors, and influence complexity metrics. A regulation indicator is not included in this dataset.

## Ethics Statement

The authors have read and follow the ethical requirements for publication in Data in Brief and confirm that the current work does not involve human subjects, animal experiments, or any data collected from social media platforms.

## Credit Author Statement

**Krzysztof Raczyński:** conceptualization, methodology, software, validation, formal analysis, investigation, data curation, writing—original draft preparation, writing—review and editing, and visualization. **Katarzyna Grala:** validation, data curation, writing—original draft preparation, writing—review and editing, and visualization. **John Cartwright:** validation, resources, data curation, writing—original draft preparation, writing—review and editing, supervision, project administration, and funding acquisition.

## Data Availability

United States Time-varying Streamflow and Fractal Dynamics Dataset (Original data).
